# A comparison of methods to determine chimpanzee home-range size in a forest–farm mosaic at Madina in Cantanhez National Park, Guinea-Bissau

**DOI:** 10.1007/s10329-019-00724-1

**Published:** 2019-04-13

**Authors:** Wilson F. Vieira, Chris Kerry, Kimberley J. Hockings

**Affiliations:** 1 Centre for Research in Anthropology (CRIA NOVA FCSH), Lisbon, Portugal; 2 Faculty of Social Sciences and Humanities, New University of Lisbon, Lisbon, Portugal; 3 Environment and Sustainability Institute, University of Exeter, Penryn, Cornwall UK; 4 Centre for Ecology and Conservation, University of Exeter, Penryn, Cornwall UK

**Keywords:** Primate home-range analysis, Chimpanzees, Anthropogenic habitats, Habitat fragmentation, Human–wildlife interactions

## Abstract

Human activities impact the distribution of numerous species. Anthropogenic habitats are often fragmented, and wildlife must navigate through human-influenced and ‘natural’ parts of the landscape to access resources. Different methods to determine the home-range areas of nonhuman primates have not considered the additional complexities of ranging in anthropogenic areas. Here, using 6 months of spatial data on the distribution of chimpanzee presence (feces, feeding traces, nests, opportunistic encounters; *n *= 833) collected across the wet and dry seasons, we examine different analytical techniques to calculate the home-range size of an unhabituated chimpanzee (*Pan troglodytes verus*) community inhabiting a forest–farm mosaic at Madina, Cantanhez National Park in Guinea-Bissau. The minimum convex polygon method and the grid cell (500 m × 500 m cell size) method estimated the chimpanzees home-range size at 19.02 and 15.50 km^2^, respectively with kernel analysis calculating a lower value of 8.52 km^2^. For the grid cell method, home-range estimates varied with cell size, with larger cells producing larger estimates. We compare our home-range estimates with other chimpanzee research sites across Africa. We recommend the use of kernel analysis for determining primate home ranges, especially for those groups exploiting fragmented habitats including forest–farm mosaics, as this method takes account of inaccessible or infrequently used anthropogenic areas across the complete home range of the primate group. However, care must be taken when using this method, since it is sensitive to small sample sizes that can occur when studying unhabituated communities, resulting in underestimated home ranges.

## Introduction

An animal’s home range is an area used to forage for food, search for mating partners, and raise offspring (Burt 1943). Home-range sizes can fluctuate over time (seasonally or yearly) depending on environmental conditions (e.g., climate) and habitat type, among other things (Newton-Fisher 2003; Wingfield 2005). Home-range size can also be influenced by anthropogenic changes to the environment and human presence. For example, habitat loss and fragmentation have reduced movements in numerous mammal species worldwide (Tucker et al. 2018). The abundance of food items in certain anthropogenic landscapes (e.g., urban or agricultural habitats) has led to some species reducing their home ranges due to high resource abundance and lack of natural competitors [e.g., raccoon (*Procyon lotor*): Prage et al. (2004); Badger (*Meles meles)*: Šálek et al. (2015)]. However, some anthropogenic disturbance factors (e.g., commercial logging and road networks) reduce natural food availability and drive some species to increase their home range to meet their nutritional needs [e.g., woodland caribou (*Rangifer tarandus caribou*): Leblond et al. (2013); black grouse (*Tetrao tetrix*): Baines and Richardson (2007)].

Some of the most threatened nonhuman primate (hereafter primate) species inhabit fragmented forest habitats that are impacted by human activities (Estrada et al. 2017). It is crucial that scientific research examines how habitats are altered by humans (including changes to food supply) and how primates respond (including how their home ranges are impacted), to implement long-term and effective conservation measures (McLennan et al. 2017). In primate species, home-range size is positively associated with body size, with omnivores and frugivores generally having larger home ranges than folivores (Milton and May 1976). Additionally, for a given species, home-range size varies inversely with habitat quality and food abundance [e.g., *Cebus capucinus*: McKinney (2011); *Macaca fascicularis*: Sha and Hanya (2013)]. On the other hand, due to the accessibility of energy-rich food resources and a lack of competitors, primate home ranges are often smaller for groups inhabiting agricultural-forest mosaics than those in more intact habitats [e.g., *Cercopithecus albogularis labiatus*: Nowak et al. (2014); *Chlorocebus pygerythrus*: Saj et al. (1999); *Papio cynocephalus*: Altmann and Muruthi (1988); *Papio ursinus:* Hoffman and O’Riain (2011); *Pongo abelli*: Campbell-Smith et al. (2011a, b)].

Chimpanzees (*Pan troglodytes*) are behaviorally flexible and are able to exploit human-influenced habitats (Hockings et al. 2012; 2015; McLennan and Hill 2012; McLennan et al. 2017; Sousa et al. 2011). They are large-bodied primates with broad diets that consist mostly of ripe fruit, but also include other plant parts in their diet, such as leaves, pith, and flowers, plus both vertebrate and invertebrate prey (Goodall 1986; Morgan and Sanz 2006; Pruetz 2006; Stanford and Nkurunungi 2003; Tutin et al. 1997; Watts et al. 2012; Wrangham et al. 1991). Chimpanzees inhabiting forest–farm mosaics often incorporate cultivated foods as part of their feeding strategy [e.g., Bossou in Guinea: Hockings et al. (2009); Bulindi in Uganda: McLennan (2013); Cadique-Caiquene in Guinea-Bissau: Bessa et al. (2015)]. Chimpanzees live in social groups (“communities”) characterized by high fission–fusion dynamics, forming temporary parties that vary in size, composition, and duration (Goodall 1986; Lehmann et al. 2007; Wrangham 1986) with individuals sharing a common home range. This social strategy allows them to respond quickly by adjusting party sizes to environmental change, including anthropogenic processes (Hockings et al. 2012; Lehmann and Boesch 2004).

Despite numerous studies of chimpanzee flexibility in response to humans and their activities (see, Hockings et al. 2015), little is known about home-range sizes in chimpanzee communities inhabiting anthropogenic habitats. Chimpanzees inhabiting farm–forest mosaics might have smaller home ranges compared to those inhabiting more intact forests as: (1) energy-rich agricultural crops, which are clumped in distribution, might minimize travel distances in search of food; and (2) roads, settlements, and large agricultural areas might act as barriers to movement. On the other hand, under certain conditions (e.g., no neighboring chimpanzee communities, and travel routes with sufficient vegetation cover), chimpanzees might range further in search of additional food resources.

Chimpanzee home-range sizes are large, on average, compared to those of most other primates (Harvey and Clutton-Brock 1981), and typically fall between 10 and 40 km^2^ (Amsler 2009). However, significant variability has been found in chimpanzee home-range sizes, from 3.1 km^2^ for the Taï Middle community (lowland forest, Ivory Coast) (Herbinger et al. 2001) to 63 km^2^ for the Fongoli community (savannah habitat, Senegal) (Pruetz 2006). Various social and ecological factors, including the distribution and availability of food, predation risk, and community size and composition, affect chimpanzee ranging and home-range size (Bertolani 2013; Lehmann and Boesch 2003).

Numerous methods have been used to examine primate home-range size and distribution, including minimum convex polygon (MCP) method, grid cell (GC) method, and kernel analysis.

MCP is a simple and frequently used method to estimate home-range size (Hayne 1949; Powell 2000). It is a non-statistical method that consists of creating a polygon with internal angles lower than 180º from the GPS points, producing an empirical estimate of the home-range size (Mohr 1947). Although widely used, which enables comparisons amongst study sites, this method presents several disadvantages: (1) it is highly sensitive to the sample size, resulting in home-range estimates increasing incrementally with the number of GPS locations collected, (2) it is sensitive to “outliers”, consequently occasional excursions can significantly influence the estimated home range, (3) it assumes that the home range is a convex polygon, which includes areas unused by the animals, thus overestimating their home range (Anderson 1982; Barg et al. 2005; Börger et al. 2006; Herbinger et al. 2001; Worton 1995a).

GC is another widely used method. It superimposes a grid with a mesh of a chosen size over the area in which traces of a species are found. The area is calculated by multiplying the sum of cells that contain traces by the area of a single cell (Chapman and Wrangham 1993; Montanari 2014). In contrast to MCP, this method can reveal the intensity of utilization of areas within the home range (Bailey and Gatrell 1995; Mizutani and Jewell 1998). However, it can be challenging to select an appropriate cell size. Although large-sized cells help to identify home-range patterns, information on the internal utilization of the habitat might be lost. Conversely, smaller cell sizes can reveal areas of intense utilization, but the high variability of quadrat counts might lead to difficulties in interpretation (Bailey and Gatrell 1995; Herbinger et al. 2001; Mizutani and Jewell 1998). A large cell size of 500 m × 500 m is frequently used to examine home-range patterns in large-bodied mammals, including chimpanzees, and is also appropriate for small sample sizes (Amsler 2009; Basabose 2005; Lehmann and Boesch 2003, 2005; Mizutani and Jewell 1998; Thompson et al. 2007).

Kernel analysis is based on the concept of utilization distribution (Van Winkle 1975). It determines the probability of finding a particular individual or group at a particular location by systematically collecting GPS data on observed locations, then using these data to calculate how intensively the group or individual use each area over time. It generates isopleths (areas demarcating regions on the map with a given probability of finding individuals) that indicate specific areas that together account for a given percentage of overall home-range occupation time (Anderson 1982; Seaman and Powell 1996; Worton 1987; Worton 1995b). Thus, the main function of this method is the probability of finding a positional point. The area is calculated using percentage isopleths indicating the intensity of utilization of an area and uniting a set of location points into a continuous surface.

Some authors suggest that statistical methods of home-range estimation, like kernel analysis, must follow the assumption that all the locations are independent (Swihart and Slade 1985a, b), which can be overcome by sub-sampling the dataset (Herbinger et al. 2001; Newton-Fisher 2003). However, since ranging is not an independent phenomenon, subsampling can have a substantial influence on the results (e.g., changes on distribution of locations) leading to loss of important information on animals’ distribution (Barg et al. 2005; Blundell et al. 2001; Cushman et al. 2005; De Solla et al. 1999). There is an ongoing debate regarding the importance of independent locational data; however, an agreed solution to improve the accuracy of kernel analysis is to increase sample size (Amsler 2009; Bertolani 2013; Montanari 2014).

Here, we test three different spatial analytical techniques (minimum convex polygon; grid cell analysis and kernel analysis) to examine the home-range size of an unhabituated and unstudied chimpanzee community living in a forest–agricultural mosaic habitat at Madina in Cantanhez National Park, Guinea-Bissau. We then compare the home-range size of this community to that of other chimpanzee communities inhabiting different habitat types with varying degrees of anthropogenic exposure, to better understand how this species might respond to human-induced change.

## Methods

### Study Site

Cantanhez National Park (CNP) is located in the southwestern region of Tombali (northeast limit 11°22′58″, 14°46′12″W and southwest limit 11º02′18″N, 15º15′58″W) in Guinea-Bissau, covering an area of approximately 1067 km^2^ (Cantanhez National Park Management Plan 2017). There are two main seasons in this region; the dry season occurs from November to mid-May and the wet season from mid-May to October. Seven species of primate inhabit CNP: western chimpanzee (*Pan troglodytes verus*), red colobus (*Procolobus badius temminckii*), black and white colobus (*Colobus polykomos*), Guinea baboon (*Papio papio*), green monkey (*Chlorocebus aethiops sabaeus*), Campbell’s monkey (*Cercopithecus campbelli*); and Senegalese bushbaby (*Galago senegalensis*) (Da Silva et al. 2014; Gippoliti and Dell’Omo 1996; Minhós et al. 2013). From the availability of suitable chimpanzee habitat in the Tombali region (CNP, Catio and Cacine), it is estimated that there are between 376 and 2632 chimpanzees (Torres et al. 2010). CNP has an estimated total of 400 chimpanzee (Casanova and Sousa 2007; Hockings and Sousa 2012) and marked-nest analysis suggests that between 17 and 106 of these are present in four forest areas in the central-southern part of the NP (Lauchande, Cadique, Caiquene, and Madina) (Sousa et al. 2011).

In CNP, the local human density is approximately 21 people/km^2^, with a population of around 24,000 people, distributed across 110 villages (Cantanhez National Park Management Plan 2017; Hockings and Sousa 2013). Deforestation and habitat fragmentation caused by slash-and-burn agriculture and clearing fields for cashew plantations is one of the main threats to the survival of primate species living in CNP (Hockings and Sousa 2012). Despite this, CNP includes some of the most well-preserved patches of primary sub-humid forests in the country (Oom et al. 2009; Temudo 2009), along with savannah, mangroves, evergreen, and semi-deciduous forests (Catarino 2004; Gippoliti and Dell’Omo 2003). Furthermore, some local ethnic groups, including the Nalu, hold traditional protective beliefs toward chimpanzees that prevent them from being hunted (Sousa and Frazão-Moreira 2010; Sousa et al. 2014). All ethnic groups (except the Balanta) hold Islamic beliefs that prohibit the killing and eating of primates (Parathian et al. 2018).

**Fig. 1:**
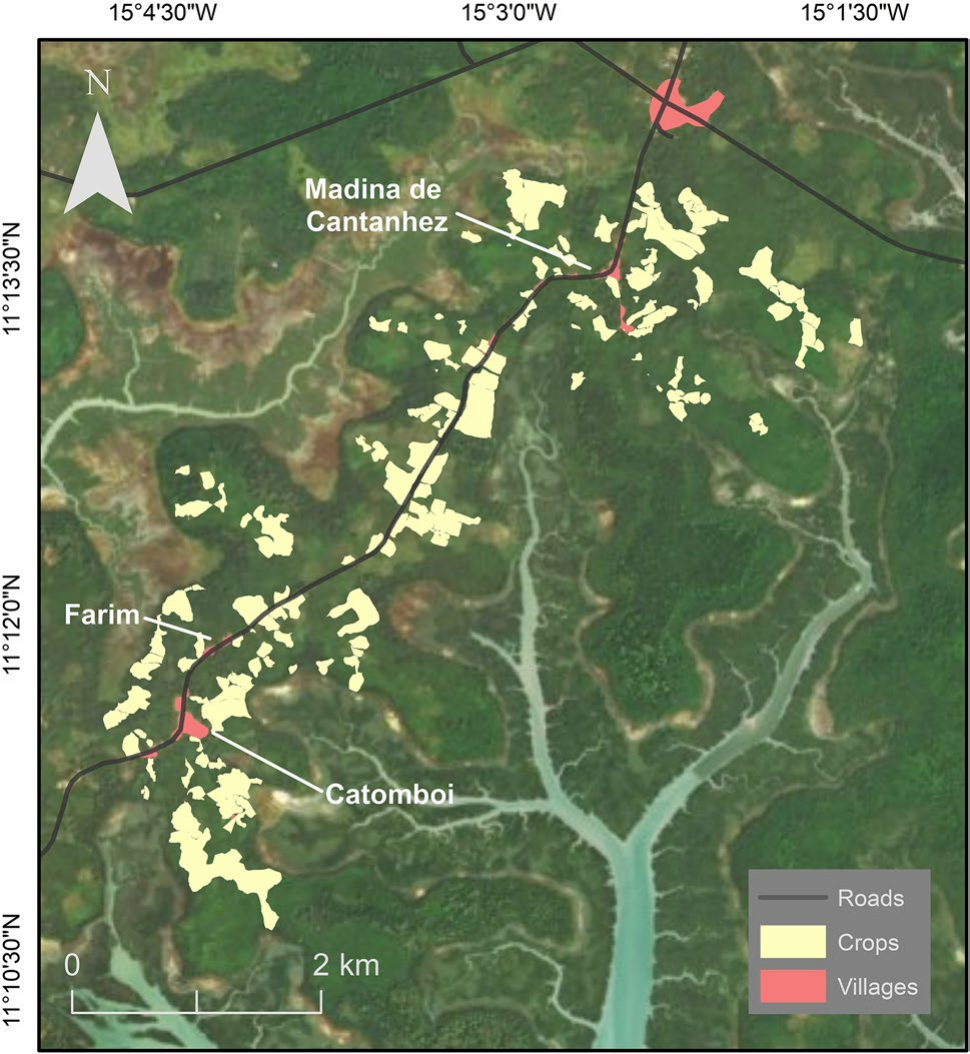
Locations of Madina de Cantanhez, Farim and Catomboi villages, cultivated areas, and main roads.* Different shades of green and blue* represent forest blocks and streams, respectively

The study area of Madina de Cantanhez (hereafter Madina), Farim and Catomboi (latitude: 11°13′N–11º19′N–11º11′N; longitude: 15 03′W–15º07′W–15º04′W) (Fig. 1) was selected based on preliminary research that estimated the location of potential chimpanzee communities in central-southern CNP (Hockings and Sousa 2013). The area is ethnically diverse: most inhabitants of Madina are Madinga or Fula, although members of other groups (e.g., Susso, Balanta) are also present, while most inhabitants of Farim and Catomboi are Nalu (Sousa et al. 2014). Based on surveys carried out during this study, there are approximately 554 inhabitants in the three villages. The surveyed area represents a highly fragmented landscape with at least 175 cultivated fields (mapped during this research) and sub-humid mature forest, secondary forest, and mangroves (Fig. 1). It is divided by a main road, which is frequently used by pedestrians and vehicles.

The Madina chimpanzee community is unhabituated to researchers. However, encounters between chimpanzees and local people occur on a daily basis especially when chimpanzees’ cross roads and enter agricultural areas or the village to feed on crops.

### Survey area and study period

**Fig. 2:**
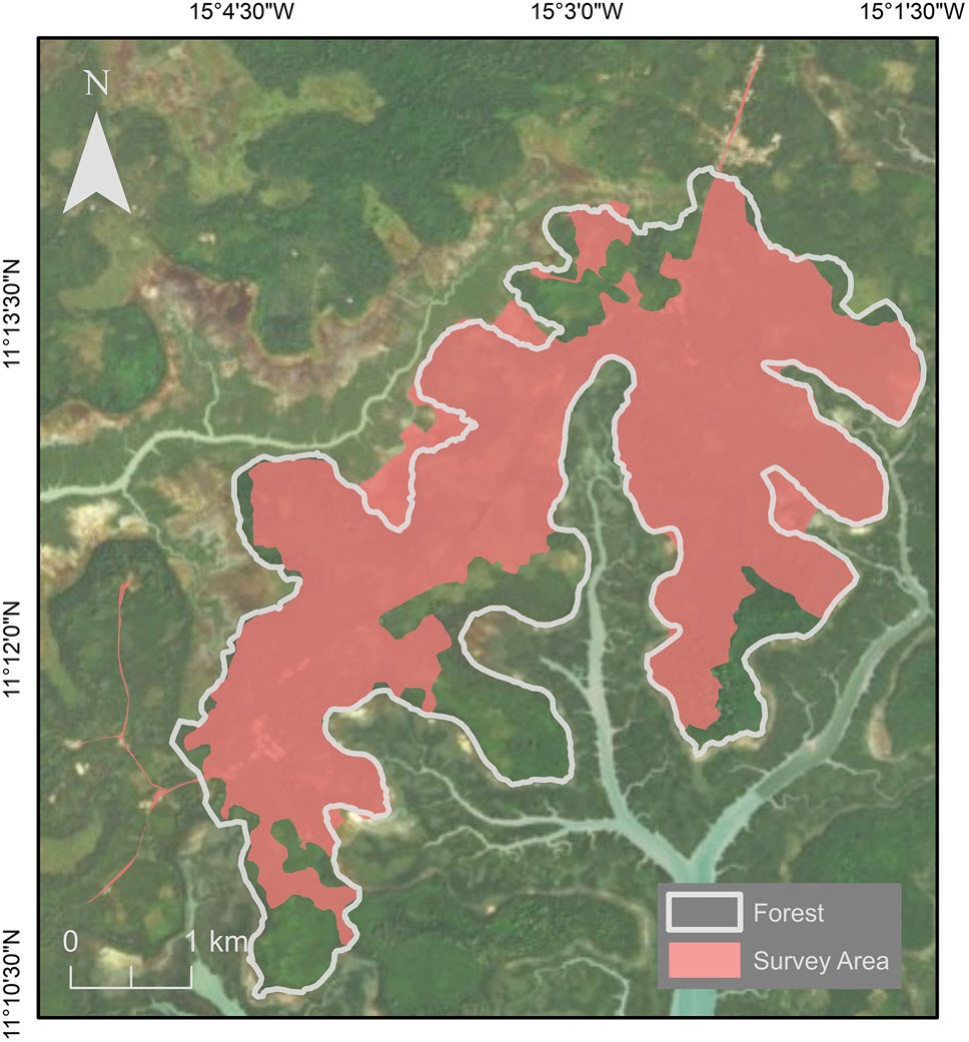
Map showing the area surveyed during this study

The survey area was chosen and delineated based on (1) knowledge acquired through our long-term presence in the area, (2) reports from local people, and (3) natural and anthropogenic barriers to the chimpanzees (e.g., forest–river interface, villages). A total area of 16 km^2^ was available to this community. We conducted recces outside of this main survey area and no chimpanzee signs were found. We surveyed 13 km^2^ of this area looking for direct and indirect signs of chimpanzee presence (Fig. 2). Of the surveyed area, 3.3 km^2^ is classified as anthropogenic including villages and cultivated fields, and 9.7 km^2^ is classified as ‘natural’, including mature forest, young forest, palm grove, and mangrove (Catarino et al. 2012; Fig. 1).

Human impacts on the landscape vary along a continuum (Hockings et al. 2015) and areas now under forest cover have been subjected to past use by people (e.g., for slash-and-burn farming). However, at the time of our study, chimpanzees could range in villages and cultivated areas (i.e., extensively modified) as well as areas covered by mature forest (i.e., not extensively modified). Below, we refer to these two general landscape types as “anthropogenic” and “natural”, respectively.

### Data collection

We collected 6 months of data over two periods (February–May and October–December 2017) including both dry and wet seasons. We marked the location of indirect signs of chimpanzee presence (i.e., feces, nests, and feeding traces) and direct encounters using a Garmin GPSMap 64 s device. We mostly found indirect signs during surveys while direct encounters were opportunistic. Due to the already fragmented habitat, we avoided clearing vegetation to establish linear transects. We followed pre-existing paths and crossed open areas of forest to search for chimpanzee signs. A local research assistant with training in identification of wildlife traces assisted with the research.

Chimpanzee feces are usually found on chimpanzee trails, under fresh nests or in recently used areas (McGrew et al. 1988). The feces of this species are distinguishable from those of other primate species by size, color, form, and odor (Bessa et al. 2015; McLennan 2013). When we found several feces in the same area (usually along trails), which we considered to be from the same individual or party (e.g., same color and age), we marked it as a single GPS location to avoid over-estimation (McLennan 2013).

We collected data from feeding traces (e.g., fruit remains, marks on tree bark) only when we were certain that the trace belonged to chimpanzees. On most occasions, we confirmed a sign to belong to chimpanzees whenever they were associated with other evidence of chimpanzee presence (e.g., feces, knuckle prints), and when it was associated to species-specific signs (e.g., fruit wadge, tool use), or whenever it was known that an individual or a feeding party had been in the area recently (McLennan 2010; Morgan and Sanz 2006; Pruetz 2006). We recorded feeding traces that we considered to be recent (i.e., less than 3 days old) and that were not in an advanced stage of decay, except when found in a new area. This ensured that we recorded all confirmed traces found in areas not frequently exploited by the chimpanzees. For each feeding trace, we marked a GPS location. When we found several feeding traces of the same age and in the same location that could be associated with larger feeding parties, we recorded only one GPS location for independence of data (McLennan 2010).

When we located a new chimpanzee nest, we searched the surrounding area to identify nest clusters (i.e., a group of nests of the same age that lie within a radius of 30 m) (Furuichi et al. 2001). If those nests were fresh, they were likely to have been built by a chimpanzee party on the same night. For each identified cluster, we marked a single GPS point. Care was taken to not sample the same nest repeatedly by using previous GPS points to identify recorded trees.

Encounters with chimpanzee individuals were mainly opportunistic. When we heard a chimpanzee party or whenever we knew the location of the party (e.g., through local reports), we would follow signs left by the chimpanzees. We only approached chimpanzees when we could ensure that we would cause minimal disturbance. We marked the GPS location of each encounter.

### Data analysis

Home-range calculations with MCP and GC used 100% of the data points collected while kernel analysis used 95% of the data points (Herbinger et al. 2001; Newton-Fisher 2003; Powell 2000; Wrangham and Smuts 1980). To calculate core areas, we used 50%, 75%, and 80% of the data points. For MCP analysis, we created polygons using the mcp function from the ADEhabitatHR package in Rstudio with Rv3.4.3 software. We calculated the areas of the polygon using the Calculate Geometry Tool of ArcMap 10.2.2. For GC analysis, we used the Create Fishnet feature of the Data Management tools in ArcMap 10.2.2. to create the grid cells with 500 m × 500 m, 200 m × 200 m and 100 m × 100 m each. We created a spatial join between the grid cell and the data on chimpanzee signs. We calculated the home range by identifying all grid cells with at least one observation recorded. The core area was established by finding cells with observations above the mean number of observations from the home-range grid cells (Montanari 2014). For kernel analysis, we calculated a kernel density raster layer using the kernel density feature from the Spatial Analyst Tool in ArcMap 10.2.2. We calculated the home range using the Isopleth command with 0.95 quantiles in Geospatial Modelling Environment 0.7.3. while we calculated the core areas using 0.5, 0.75, and 0.8 quantiles.

## Results

### Home-range and core-area estimates

**Fig. 3:**
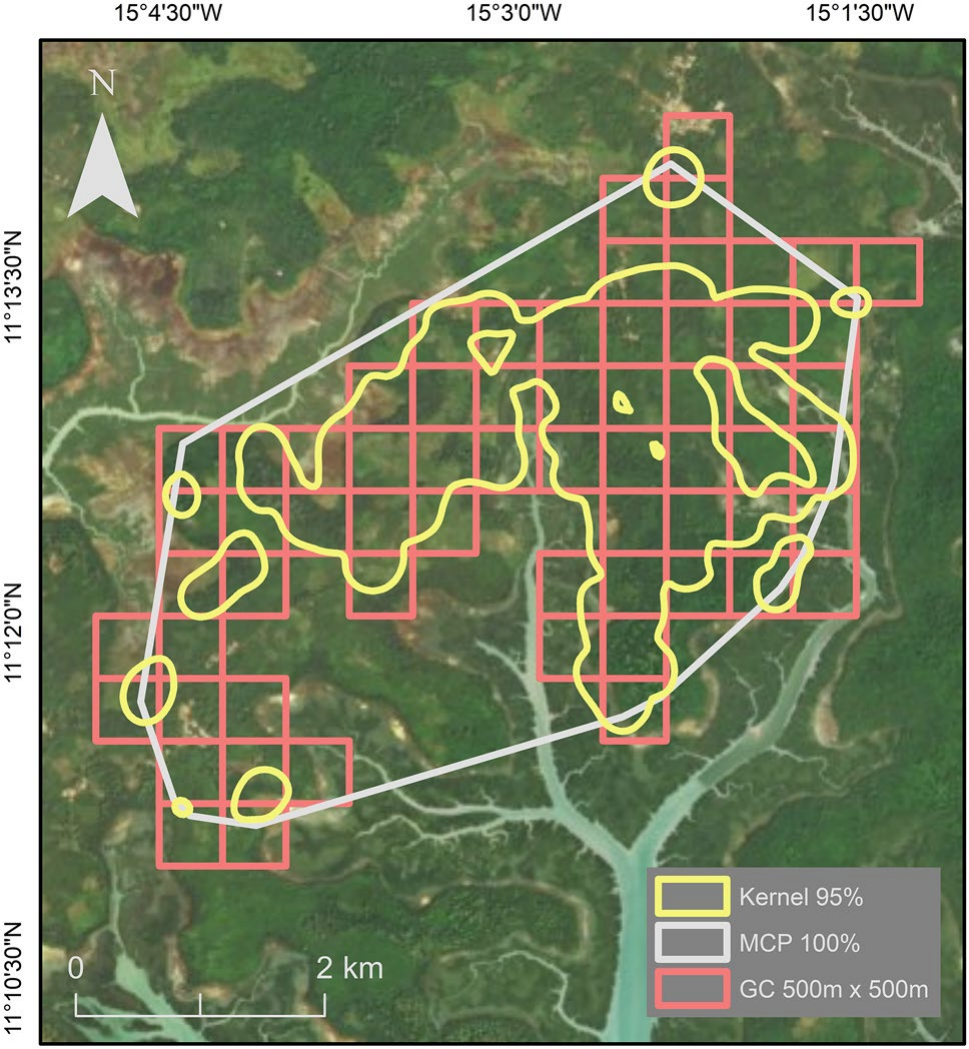
Home ranges calculated using three different methods: kernel analysis (95%), minimum convex polygon (100%), and grid cell (500 m × 500 m)

**Fig. 4:**
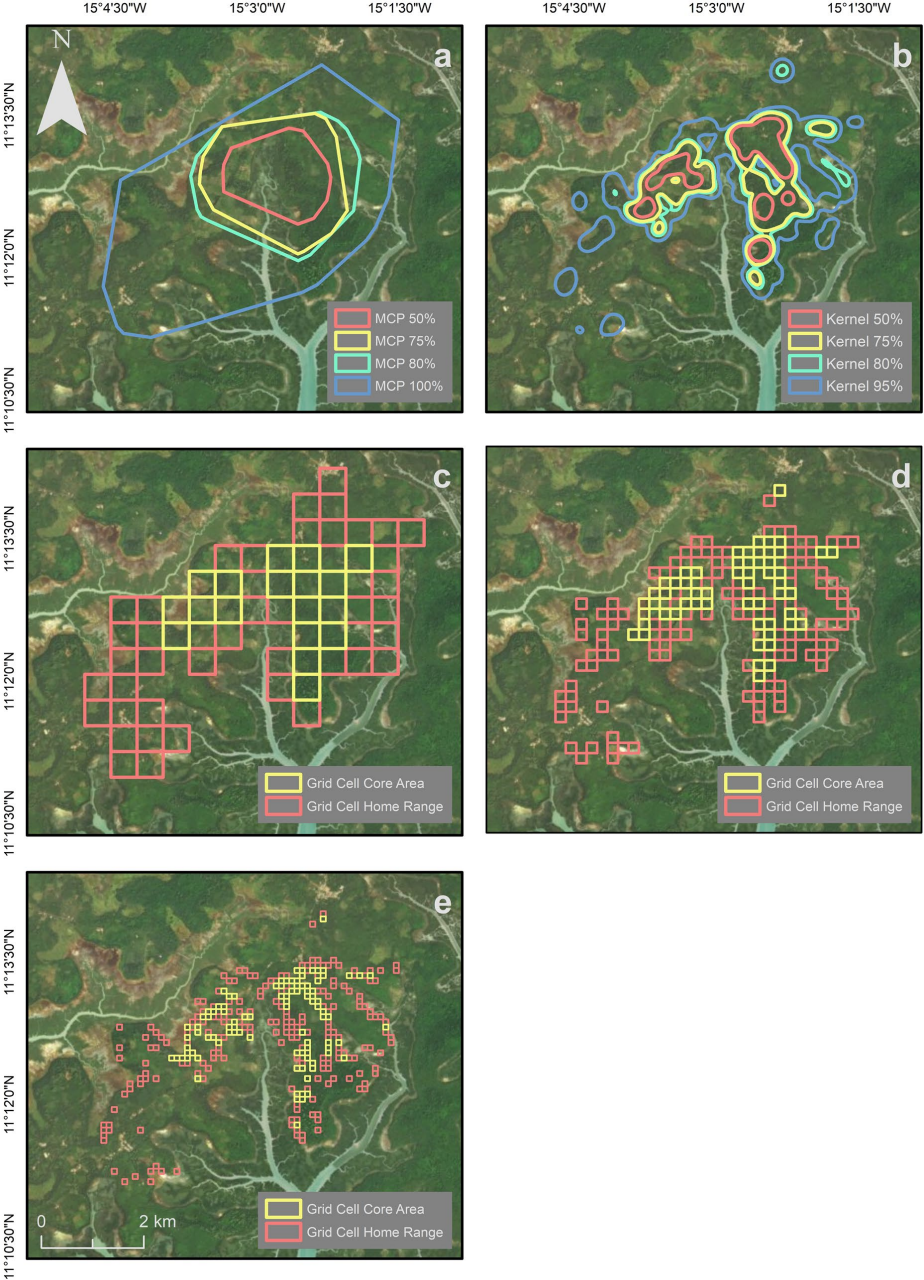
Home range and core areas calculated using **a** minimum convex polygon (100%, 80%, 75% and 50%); **b** kernel analysis (95%, 80%, 75% and 50%); **c** grid cell 500 m × 500 m; **d** grid cell 200 m × 200 m; **e** grid cell 100 m × 100 m

We found 833 chimpanzee traces across the study area in both natural and anthropogenic habitats. On the days that we found traces (*N* = 77 of 137 days), the number of traces varied with a mean of 10.8 (± SD 9.4) per day. The MCP method yielded the largest estimated home-range size, followed by the GC (500 m × 500 m) method, then kernel analysis, which estimated a home-range size of less than half that of MCP (Table 1; Fig. 3). Kernel analysis more clearly demonstrates the use of habitat fragments by chimpanzees at a finer scale than the other two methods. GC method with 200 m × 200 m and 100 m × 100 m cell size provided smaller estimates than the other methods. The home-range estimate using GC 200 m × 200 m was most similar to the estimate generated using kernel analysis (Table 1; Fig. 4). Estimates for core-area sizes followed the same rank order but vary less.

**Table 1:** Estimates of core-range areas (km^2^) and home-range size (km^2^) using 100% of data points for MCP and GC (with different sized grid cells), and 95% for kernel analysis for chimpanzees at Madina, Cantanhez NP

Method		% Data points	Area estimated (km^2^)
MCP		100	19.02
		80	6.82
		75	5.62
		50	2.72
GC	500 m × 500 m	100	15.50
		77	4.50
	200 m × 200 m	100	7.28
		73	2.20
	100 m × 100 m	100	3.05
		68	0.96
Kernel analysis		95	8.52
		80	4.30
		75	3.56
		50	1.48

### Comparison of chimpanzee study sites

The results of the home-range analysis for chimpanzees at Madina were compared with 13 other chimpanzee study sites across Africa (Table 2). Six sites were characterized as low anthropogenic exposure, four as medium anthropogenic exposure, and three as high anthropogenic exposure. For most sites, MCP or GC methods generated the largest estimates of home-range size, with smaller estimates using kernel analysis (but see Budongo and Seringbara) (Table 2). Home-range estimates using kernel analysis for habituated and unhabituated chimpanzee communities inhabiting predominantly forested areas averaged 10.4 km^2^ (range, 3.1–19.5, *N* = 6 habituated communities). Chimpanzees at Madina have a slightly smaller-than-average home-range size of 8.5 km^2^ compared to other chimpanzee communities.

**Table 2:** Home-range sizes of 14 chimpanzee communities, including Madina (adapted from Bertolani 2013)

Study site	Study period	Country	Anthrop. exposure	Level of habituation	Comm. size	Home range (km^2^)				References
						MCP	GC	K95/FT95*	Other	
Budongo	1994–1995	Uganda	Med	Habituated	46	6.8	–	6.9	–	Newton-Fisher 2003
Gombe	1975–1992	Tanzania	Med	Habituated	51	11	–	–	–	Williams et al. 2004
Tai North	1996–1997	Ivory Coast	Low	Habituated	35	16.8	18.3	7.5*	–	Herbinger et al. 2001
Tai South	1996–1997	Ivory Coast	Low	Habituated	63	26.5	23.3	9.5*	–	Herbinger et al. 2001
Tai Middle	1996–1997	Ivory Coast	Low	Habituated	11	12.1	13	3.1*	–	Herbinger et al. 2001
Ngogo	2003–2006	Uganda	Med	Habituated	143	27.7	29.3	19.5	–	Amsler 2009
Bulindi	2006–2008 (15 months)	Uganda	High	Semi-habituated	25 (est)	21	–	–	–	McLennan 2010
Kanyawara	2007–2009	Uganda	Med	Habituated	48	27.4	26	16.2	–	Bertolani 2013
Seringbara	2012––2013 (1 year)	Guinea	Low (est)	Unhabituated	100 (est)	29	20.5	35.7^a^	–	Montanari 2014
Madina	2017 (6 months)	Guinea-Bissau	High **(**est)	Unhabituated	–	19.2	15.5	8.5	–	This study
Kahuzi	1994-2000	DR Congo	Low	Semi-habituated	23	–	12.8	–	–	Basabose 2005
Fongoli	2001–2004	Senegal	Low	Semi-habituated	32	–	–	–	63	Pruetz 2006
Cadique–Caiquene	2013 (9 months)	Guinea-Bissau	High (est)	Unhabituated	35 (est)	–	–	–	7.9	Bessa et al. 2015
Bossou	1995 (13 months)	Guinea	High	Habituated	20	–	–	–	15	Yamakoshi 1998

Studies employed different methods to calculate home-range size, including minimum convex polygon (MCP), grid cell (GC), kernel (K) analysis, and Fourier’s transformation (FT). For the GC method, studies used 500 m × 500 m cells size, with exception of Kahuzi Biega, which used 250 m × 250 m-sized cells. All sites are classified as predominantly moist forest except for Kahuzi Biega (montane forest) and Fongoli (savanna-woodland). Degree of anthropogenic exposure was categorized as low, medium, or high according to site disturbance scores for long-term research sites (as reported in Hockings et al. 2015). Based on ratings of four different disturbance variables where one represents minimum disturbance and four represents maximum disturbance for each variable (i.e., disturbance scale of 4–16), we classify low as from 4 to 7 points, medium as 8–11 points, and high as 12–16 points. Where sites are not included in analyses by (Hockings et al. 2015), we estimate anthropogenic exposure levels based on information presented in the associated research article. We include reported habituation levels. Mean community size is given for studies covering multiple years (as per Bertolani 2013) and estimated community sizes of unhabituated communities are labeled

^a^Although not specified in Montanari (2014), this high value could be due to the choice of smoothing parameter which is least squares cross validation (i.e., a calculation for how big each cell is within the kernel and how neighboring cells influence the focal cell). If there were few data, this could have resulted in large cell sizes and stretching of the data, especially if data points were skewed towards the edge of the territory

* Fourier's transformation

## Discussion

The methods (MCP, GC, and kernel analysis) we used to calculate the Madina chimpanzees’ home-range size produced different estimates. However, the core-range area estimates varied less. MCP method revealed an area twice the size of that calculated using kernel analysis. GC results varied according to the cell size used. Using a cell size of 500 m × 500 m, the estimated home-range area was similar to estimates generated through MCP. However, 200 m × 200 m and 100 m × 100 m cell sizes generated much smaller home-range estimates. Like most home-range estimates for other chimpanzee communities, at Madina kernel analysis revealed the smallest area estimates compared to other methods, except when smaller cell sizes were used for the GC method.

The landscape inhabited by chimpanzees at Madina is a mosaic of roads, villages, and cultivated fields, as well as rivers and small streams that surround the forest, giving it an irregular shape. MCP method incorporates anthropogenic and natural areas that chimpanzees cannot use. By using the peripheral points to create a polygon that represents the community’s territory, MCP overestimates home-range size. These “outliers” will have a strong effect on the home-range estimate by “stretching” the polygon (Barg et al. 2005; Harris et al. 1990). This issue can be overcome by eliminating the “outliers” and recalculating the home range with a given percentage of the original dataset (Mizutani and Jewell 1998; Worton 1987; Worton 1995a), creating an area of high-density GPS locations (core areas). However, MCP cannot calculate core areas because it does not statistically identify areas of high levels of utilization.

The variation in cell size used in GC analysis influenced the overall home-range size of the chimpanzee community (Table 1). Like MCP, GC is also likely to include areas not utilized by the chimpanzees, especially with large cell sizes. Cells of 500 m × 500 m are commonly used in chimpanzee studies (Amsler 2009; Lehmann and Boesch 2005; Herbinger et al. 2001). However, considering the fragmented and mosaic landscape at Madina, this cell size is too large. The smallest cell size used in this research (100 m × 100 m) is ideal for determining areas of high utilization by the chimpanzees but makes it difficult to identify a home range. The 200 m × 200 m cell size seems most appropriate since it includes fewer areas that are not used by the chimpanzees (compared to 500 m × 500 m cells), but cells outside of core areas are more likely to be connected allowing better visualization of home ranges (compared to 100 m × 100 m cells).

Kernel analysis has been proposed as the most accurate method to calculate home-range sizes (Seaman and Powell 1996; Worton 1987, 1989, 1995b), although issues of small datasets and the independence of data points must be carefully considered (Bertolani 2013). In this study, we suggest that MCP and GC using large-sized cells of 500 m × 500 m might be inappropriate for understanding primate home ranges in anthropogenic mosaic landscapes. Although we show that smaller-sized cells can better represent chimpanzees’ home ranges, we do not advise the use of this method due to the subjectivity of choosing the “ideal” cell size for a given habitat. Kernel analysis more accurately represented the locations of traces found during this study and hence provides a more realistic estimation of the home-range size of this community. This research recommends that the home range of the Madina chimpanzees is taken as approximately 8.5 km^2^ with a core area (using kernel analysis of 75% of the data points) of 3.6 km^2^.

We recognize some issues that may influence our results. Increasing sample size maximizes the reliability of kernel analysis (Amsler 2009; Montanari 2014). Our results rely on 833 chimpanzee locations collected on 77 days (although the overall study period was longer). This small sample size may have led to an underestimation of the chimpanzees’ home-range area. A longer study period would ultimately increase the amount of data collected and the accuracy of estimations. Approximately 3 km^2^ of the forest at Madina (which the chimpanzees were likely to use) was not surveyed due to inaccessibility. Complementary data collection methods (e.g., camera–traps) might solve this problem and ensure all parts of the landscape are included in home-range estimates.

The home range of the Madina chimpanzee community was compared to 13 other studies of chimpanzee home-range areas across Africa. Although three sites (Caiquene-Cadique, Bulindi, and Bossou) provide information on home-range size for chimpanzees inhabiting sites with high human impact, kernel analyses were not used. This makes comparisons difficult. The home-range estimate for Madina is similar to some other sites including Budongo, Tai North, and Tai South (Table 2). The use of different analytical tools and environmental factors might limit the reliability of comparisons between study sites (De Luca et al. 2010; Kolodzinski et al. 2010; Newton-Fisher 2003). Different factors, such as sampling effort, level of habituation to human observers, and study length, make comparisons between sites problematic (Börger et al. 2006). Also, categorizing the degree of anthropogenic exposure at any site is complex, and our measure of anthropogenic exposure (as taken from Hockings et al. 2015) is crude. Different sites will have historically been impacted and continue to be impacted by human activity in different ways, which will affect chimpanzee ranging. Without a standardization of methods to estimate home-range size across chimpanzee sites, the influence of environmental and anthropogenic factors on chimpanzee distribution is difficult to predict in the face of increasing human pressure. This is important, as increasing human populations and human activity across tropical Africa will continue to force chimpanzee communities into more fragmented and disturbed habitats.
